# Temporal Dynamics of Incidence of Shot Hole Disease Affected by Training Systems and Cultivar Susceptibilities in an Integrated Plum Orchard

**DOI:** 10.3390/jof8060580

**Published:** 2022-05-28

**Authors:** Bianka Molnár, Szilárd Szabó, Imre J. Holb

**Affiliations:** 1 Faculty of Agronomy, University of Debrecen, Böszörményi út 138, H-4032 Debrecen, Hungary; molnarbianka@agr.unideb.hu; 2 Department of Physical Geography and Geoinformatics, University of Debrecen, H-4032 Debrecen, Hungary; szabo.szilard@science.unideb.hu; 3 Eötvös Loránd Research Network (ELKH), Centre for Agricultural Research, Plant Protection Institute, H-1022 Budapest, Hungary

**Keywords:** shot hole, *Stigmina carpophila*, *Wilsonomyces carpophilus*, training system, cultivar susceptibility, plum cultivar, disease incidence, AUDPC, temporal dynamics

## Abstract

Shot hole disease (SHD) can cause severe epidemics in plum orchards, depending on cultivar susceptibility and training system; however, the combined effect on the progress of temporal disease and on the possible reduction in SHD in the disease management was not investigated. The aim of this 3-year study was (i) to monitor and analyze the temporal dynamics of SHD progress under four training systems (4 × 1.5, 4 × 2, 5 × 2.5 and 6 × 3 m) and on four plum cultivars (‘Čačanska lepotica’, ‘Bluefre’, ‘Stanley’ and ‘President’) in an integrated plum orchard; (ii) to identify those time periods when training system and cultivar combinations can reduce the disease development. Both SHD incidences and the area under the disease progress curves (AUDPC) were significantly affected by the training system, cultivar and year. Plum cultivars with high or mid–high susceptibility to SHD showed continuous SHD development from May to November, while cultivars with low susceptibility to SHD showed no symptoms until mid-summer and then progressed slowly until November. High (4 × 1.5 m) vs. low (6 × 3 m) density training systems reduced SHD incidence and AUDPC consistently for three cultivars (‘Čačanska lepotica’, ‘Stanley’ and ‘President’) in September, October and November, compared to the high-density training system. Only cv. ‘Bluefre’ showed no effect either on disease incidence or AUDPC, due to very high disease incidences in all training systems from September to November. In conclusions, combinations of training system and cultivar can significantly reduce SHD incidence, which may be successfully used as a part of the integrated pest management approach during the establishment new plantations.

## 1. Introduction

The fungus of *Stigmina carpophila* (syn. *Clasterosporium carpophilum*, *Wilsonomyces carpophilus*) causes shot hole disease (SHD) in most stone fruit orchards, including plum [1,2,3,4,5].

Symptoms of SHD occur on the leaves, shoots and fruits of most cultivated stone fruit species [6]. In the case of plum, the leaf symptom of SHD is the most common symptom type [7,8,9,10]. Leaf symptoms appear as tiny light spots that gradually turn brown. Later, a purple-brown border develops around the spots. The middle of the spots die and fall out and the ‘shot hole’ symptom appears [11,12,13]. Under favorable weather conditions, SHD becomes severe and the leaves of the tree fall before harvest, resulting in an early defoliation of the tree [14]. Due to early leaf fall, the health of trees reduces year by year which is also reflected in yield reductions [6,15,16,17,18].

Management of SHD usually requires 1 to 3 sprays during flowering then an additional spray after fruit set [17,19,20]. In the case of severe infection, copper sprays are recommended at leaf fall in autumn [21]. Due to environmental concerns and chemical control compounds’ detrimental effects on human health, interest has largely increased regarding environmentally friendly methods to control SHD, such as the biological control agents of the strain OSU-142 of *Bacillus subtilis* in the biological control of SHD [20].

Knowledge on plant disease epidemics is of great help in designing and implementing successful disease management [22,23]. This is especially essential for the foliar fungal diseases of fruit tree species as they cause early defoliations and, therefore, successful disease control is essential not only for annual yield but for the lifespan of the tree. The temporal progress of the foliar fungal diseases of fruit tree species were investigated; for instance, for cherry vs. leaf spot [24] and peach vs. shot hole pathosystems [25]. However, detailed temporal disease progress was not performed for SHD on plum cultivars under various training systems.

Cultivar susceptibility and training system affect the fruit disease epidemics of fruit species [6]. Cultivar susceptibility to SHD was studied in several fruit tree species, such as almonds [11], peaches [25], apricots [26,27], nectarines [28,29] and plums [7]. In plum studies, cultivars showed various susceptibility to SHD [7,30,31]. The study of Bubici et al. [7] showed that cv. ‘Golden Plumza’ was highly susceptible to SHD, while cvs ‘Angeleno’; ‘Autumn Giant’; ‘Fortune’; ‘Green Sun’; ‘October Sun’; ‘Santa Rosa’; ‘Sorrisodi Primavera’; and ‘T.C. Sun’ showed intermediate reactions to shot hole disease. Brózik and Kállay [31] revealed that plum cultivars ‘President’ and ‘Stanley’ are susceptible, while cvs ‘Bluefre’ and ‘Čačanska lepotica’ are considered moderately susceptible to SHD. Benedek et al. [30] showed that plum cultivars ‘Čačanska lepotica’; ‘Debreceni muskotály’; ‘Olasz kék’; ‘Čačanska najbolja’; and ‘Nagy Zöld Ringló’ are susceptible to SHD. Plum cultivars’ degree of resistance to SHD is unknown. The effect of a training system on cultivar reactions to SHD was not investigated previously, despite the fact that cultivar susceptibility in various training systems can result in reducing the temporal progress of fungal diseases on leaves.

The aim of this three-year study was to (i) monitor and analyze the temporal dynamics of SHD progress under four training systems and on four plum cultivars in an integrated plum orchard; (ii) identify those time periods when training system and cultivar combination can reduce the disease development.

## 2. Materials and Methods

### 2.1. Orchard Site, Plant Material, Expermental Desingn and Orchard Management

A three-year study (2017, 2018, and 2019) was performed in an integrated plum orchard in Eastern Hungary. The orchard was established at the University of Debrecen Experimental Station, Debrecen-Pallag (47°31′60″ N, 21°37′60″ E) in the spring of 1997 with 4 plum cultivars (‘Čačanska lepotica’, ‘Bluefre’, ‘Stanley’ and ‘President’). Cultivar characteristics including pedigree, origin, susceptibility to SHD and harvest time are given in Table 1. The trees were grafted on myrobalan ‘C 359’ rootstock. The trees of each cultivar were planted in four training systems containing a 0.25 ha plot of each cultivar. The four training systems were designed as high, mid, low to mid, and low densities with tree spacings of 4 × 1.5, 4 × 2, 5 × 2.5 and 6 × 3 m, respectively. The trees were pruned to slender spindle for the training system that was spaced at 4 × 1 m (to free the spindle for the training systems that were spaced at 5 × 2.5 and 6 × 3 m) and to a combination of slender and free spindle for the training system that was spaced at 4 × 2. The experimental design was a split plot, where the three years were referred to as blocks, the four training systems as main plots (replicated four times) and the four cultivars as subplots.

The orchard soil type was Lamellic-Brunic Arenosol soil with alternating layers of clay [32]. Bare soil was maintained mechanically with a distiller in the spacings between rows, and 0.5 m wide straw mulch was used in the rows. Tree pruning, nutritional management and spray schedules against shot hole disease were prepared according to the integrated fruit production guidelines.

Trees in the high and mid training systems received an annual summer pruning in July, and a supplementary winter pruning was performed every 2nd and 3rd year for removing the twig part of the trees that were older than 4 years. Trees in the low to mid and low training systems received a winter pruning in March of each year and no summer pruning was performed.

The orchard relied on the annual application of a nitrogen–phosphorus–potassium (NPK) complex fertilizer (Péti Kevert NPK Műtrágya, Nitrogénművek GmbH, Pétfürdő, Hungary) at the beginning of March at a dosage of 100 kg ha^−1^ active ingredient with 10:15:15 N–P–K ratio for nutrient supply. The orchard was not irrigated.

Sprays against SHD started at the dormant bud stage; copper hydroxide was used (0.1%; Funguran-OH 50 WP, 77%, Spiess-Urania Chemicals GmbH, Hamburg, Germany) and then additional sprays were applied during the season with fungicide active ingredients of: penconazole, tebuconazole, prochloraz, mancozeb, captan and copper hydroxide from mid-April (white flower bud) to the end of September (after harvest) (Table 2). All the sprays were applied with a Kertitox 2000 axial blower spray machine (Debreceni Gépgyár B.V., Debrecen, Hungary) with a ceramic hollow cone at 1.1–1.2 MPa with a volume of 1000 L ha^−1^.

### 2.2. Meteorological Data

During the 3-year periods, a Metos Compact agrometeorological station (Pessl Instrument GmbH, Weiz, Austria) was operated to measure rainfall (mm day^−1^) and the mean daily temperature (°C day^−1^) from 15 April to 15 October in 2017, 2018 and 2019.

### 2.3. Shot Hole Disease Assessment

Disease assessments were performed in the middle 10 trees of each cultivar subplot in each year for the four cultivars and four training systems. A total of 4 × 100 leaves were assessed in each tree, thus the trees were divided into four quadrants. The presence of SHD on the leaves of each quadrant were determined in each year for the four cultivars and four training systems. Seven assessments were conducted in each year on the first decade of May, June, July, August, September, October and November. A leaf was considered diseased if at least one visible SHD lesion was observed. SHD incidence was calculated as values from the quadrants were averaged to obtain the percentage of diseased leaves.

### 2.4. Data Analyses

SHD incidences from the four replicates were averaged to obtain a single value for each year, training system, cultivar and assessment date. In addition, SHD incidences of the last assessment date (final SHD incidence—*Y_f_*) were separately analyzed with a single value for each year, training system and cultivar. Moreover, the area under the disease progress curve (AUDPC) was calculated for each year, training system and cultivar. AUDPC as percent days was calculated as:

(1) $$\mathrm{AUDPC}\;\;=\sum_{i}^{n-1}\;\left(\frac{y_{i}+y_{i+1}}{2}\right)\times \left(t_{i+1}-t_{i}\right)$$

where ‘*n*’ is the total number of assessments, ‘*y_i_*’ is SHD incidence at the ‘*i*’th assessment date and the term of $t_{i+1}-t_{i}$ is the time duration between two assessments.

Then, the SHD incidences, final SHD incidence and AUDPC data were analyzed by a split-plot analysis of variance (ANOVA) using the statistical package of Statistical Analysis System v. 8.1; SAS Institute Inc., Cary, NC. Means were separated by the least significance difference (LSD) test using LSD_0.05_ values. Significant F tests (*p* = 0.05) were followed by an LSD test for a comparison of the means of the training systems, cultivars or assessment dates using LSD_0.05_ values. Prior to the analyses, SHD incidences data were arcsine-square root transformed in order to make the data normally distributed. Standard errors and LSD_0.05_ values for the differences are given in the figures and tables as appropriate.

In order to visualize the time periods when training system and cultivar combination could reduce the disease development, significant F tests (*p* = 0.05) followed by LSD tests were prepared for each assessment date for each training system vs. cultivar combination using LSD_0.05_ values.

## 3. Results

### 3.1. Environmental Monitoring

The daily mean temperature ranged from 5.9 to 27.5 °C, 8.2 to 26.6 °C, and 8.9 to 27.3 °C in 2017, 2018, and 2019, respectively, from 15 April to 15 October. Rainfall amounts during the same periods were 279.2, 212.1, and 325.1 mm in 2017, 2018, and 2019.

### 3.2. Disease Progress

**Figure 1:**
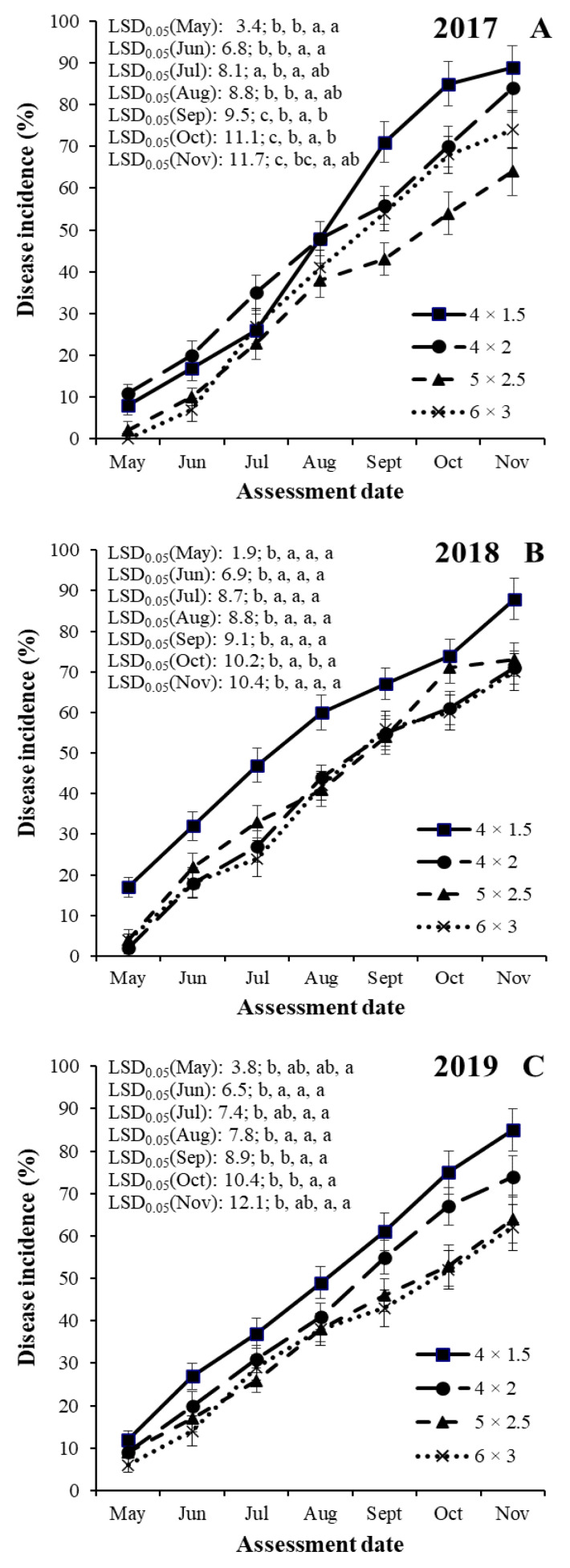
Temporal dynamics of shot hole incidence on plum cultivar ‘Čačanska lepotica’ in four training systems (4 × 1.5, 4 × 2, 5 × 2.5 and 6 × 3 m) in the years of 2017 (**A**), 2018 (**B**) and 2019 (**C**) at seven assessment dates (May, Jun, Jul, Aug, Sept, Oct and Nov) in an integrated plum orchard at Debrecen-Pallag, Eastern Hungary. Values within the given cultivar coupled with different letters are significantly different among each training system at *p* = 0.05, according to LSD *t*-tests. First, second, third and fourth letters of significance after LSD_0.05_ values correspond to training systems of 4 × 1.5, 4 × 2, 5 × 2.5 and 6 × 3 m, respectively.

**Figure 2:**
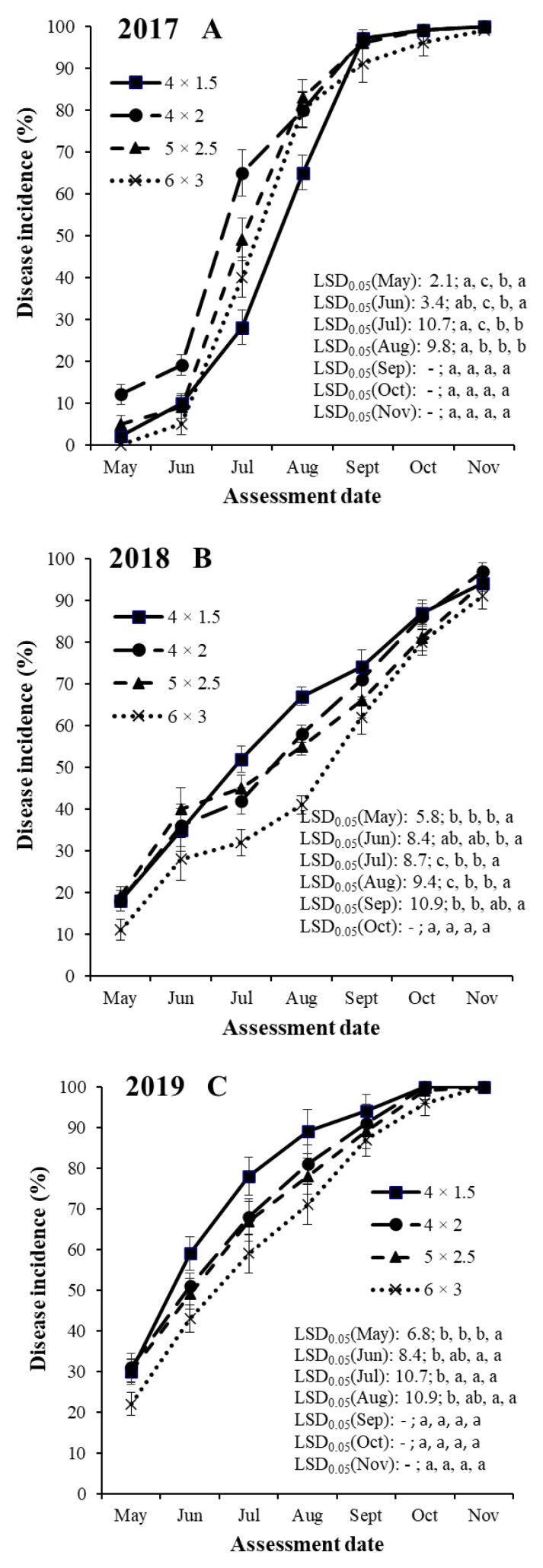
Temporal dynamics of shot hole incidence on plum cultivar ‘Bluefre’ in four training systems (4 × 1.5, 4 × 2, 5 × 2.5 and 6 × 3 m) in the years of 2017 (**A**), 2018 (**B**) and 2019 (**C**) at seven assessment dates (May, Jun, Jul, Aug, Sept, Oct and Nov) in an integrated plum orchard at Debrecen-Pallag, Eastern Hungary. Details of explanations on significant symbols and LSD-test are given in Figure 1.

**Figure 3:**
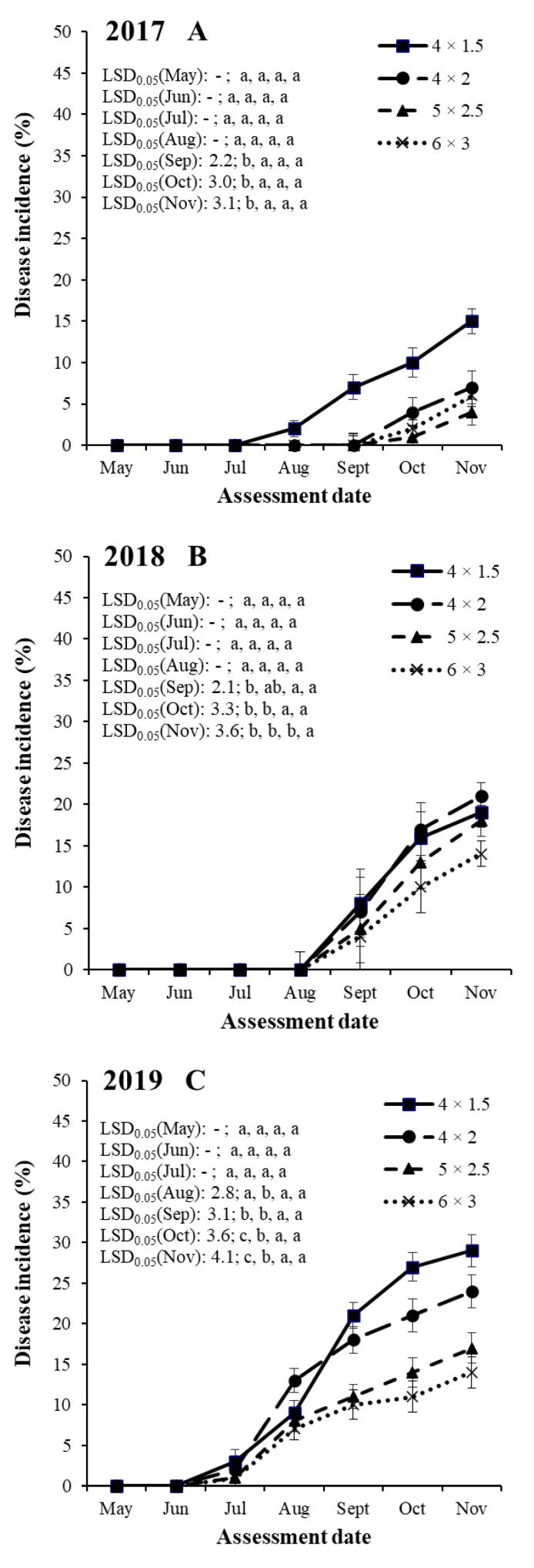
Temporal dynamics of shot hole incidence on plum cultivar ‘Stanley’ in four training systems (4 × 1.5, 4 × 2, 5 × 2.5 and 6 × 3 m) in the years of 2017 (**A**), 2018 (**B**) and 2019 (**C**) at seven assessment dates (May, Jun, Jul, Aug, Sept, Oct and Nov) in an integrated plum orchard at Debrecen-Pallag, Eastern Hungary. Details of explanations on significant symbols and LSD-test are given in Figure 1.

**Figure 4:**
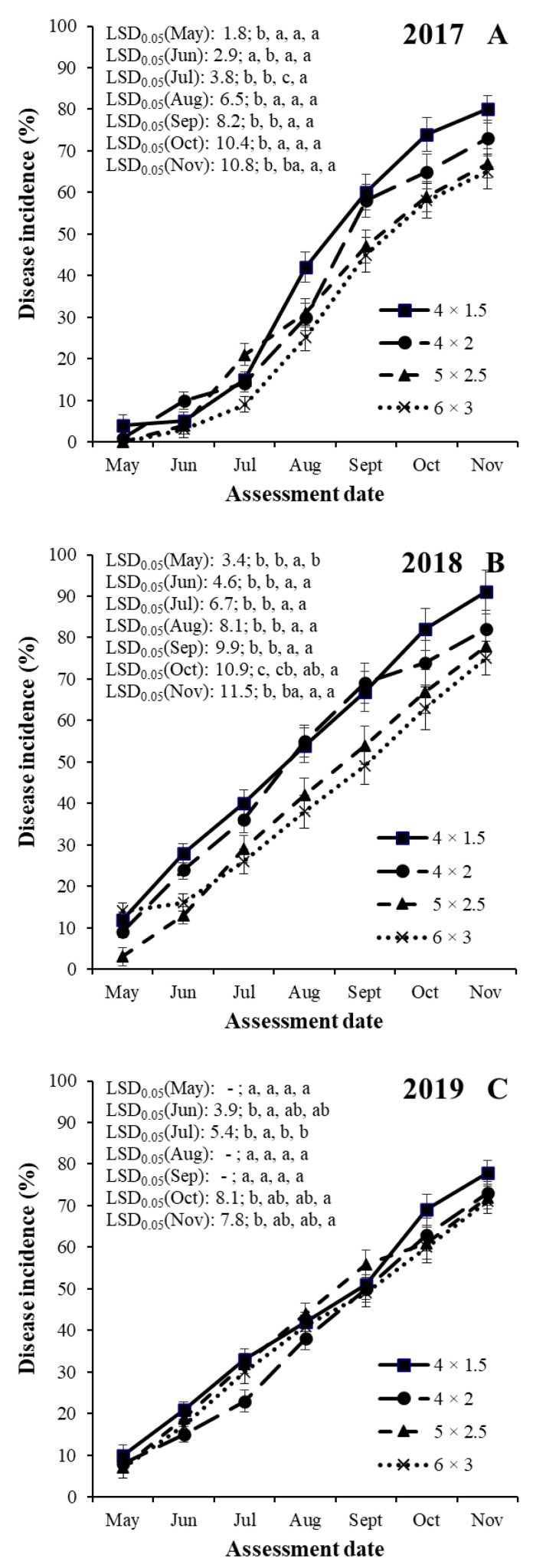
Temporal dynamics of shot hole incidence on plum cultivar ‘President’ in four training systems (4 × 1.5, 4 × 2, 5 × 2.5 and 6 × 3 m) in the years of 2017 (**A**), 2018 (**B**) and 2019 (**C**) at seven assessment dates (May, Jun, Jul, Aug, Sept, Oct and Nov) in an integrated plum orchard at Debrecen-Pallag, Eastern Hungary. Details of explanations on significant symbols and LSD-test are given in Figure 1.

An analysis of variance for SHD incidence indicated significant (*p* < 0.001) differences among years, training systems, cultivars and assessment dates (Table 3). There were no significant interactions among treatment factors. Therefore, SHD incidence data sets were shown separately for the years, training systems and assessment dates for each of the four cultivars (Figure 1, Figure 2, Figure 3 and Figure 4).

#### 3.2.1. Cultivar ‘Čačanska lepotica’

In the case of plum cultivar ‘Čačanska lepotica’, SHD incidences were the highest in 2017 in the 4 × 1.5 m training system (ranging between 8.1 and 89.4%) and the lowest in 2019 in the 6 × 3 m training system (ranging between 5.9 and 62.1%, Figure 1).

SHD progress in the four training systems started before the first assessment date (the first day of May) and increased with various progress speeds until the last assessment date (in November) in all years (Figure 1).

In 2017, the SHD incidence values were the highest in the mid-density training system (4 × 2 m) from May to July, which were significantly higher compared with the training system of 5 × 2.5 and 6 × 3 m in May and June, and in the training systems of 4 × 1.5 and 5 × 2.5 in July (Figure 1A). The SHD incidence values were the highest in the high-density training system (4 × 1.5 m) from August to November, which were significantly higher compared with the training system of 5 × 2.5 m in August, in the training systems of 4 × 2, 5 × 2.5, and 6 × 3 m in September and October, and in the training systems of 5 × 2.5 and 6 × 3 m in November.

In 2018, the SHD incidence values were the highest in the high-density training system (4 × 1.5 m) in all assessed months, which were significantly higher compared with the other three training systems (4 × 2, 5 × 2.5 and 6 × 3 m) with the exception of October when the values in the high-density training system (4 × 1.5 m) were significantly different from the values of the training systems of 4 × 2 and 6 × 3 m (Figure 1B).

In 2019, the SHD incidence values were the highest in the high-density training system (4 × 1.5 m) in all assessed months, which were significantly higher than the values in the training system of 6 × 3 m in May, in the training systems of 4 × 2, 5 × 2.5, and 6 × 3 m in June and August, and in the training systems of 5 × 2.5 and 6 × 3 m in July, September, October and November (Figure 1C).

#### 3.2.2. Cultivar ‘Bluefre’

In the case of plum cultivar ‘Bluefre’, SHD incidences were the highest in 2019 in the 4 × 1.5 m training system (ranging between 30.8 and 100%) and the lowest in 2018 in the 6 × 3 m training system (ranging between 11.1 and 90.8%, Figure 2).

SHD progresses in the four training systems started before the first assessment date (first decade of May) except for the training system of 6 × 3 m in 2017 (Figure 2). In 2017 and 2019, the disease progress rapidly increased until September when it levelled off (Figure 2A,C). In 2018, SHD incidences increased with various progress speeds until the last assessment date (in November) in all years (Figure 2B).

In 2017, the SHD incidence values were the highest in the mid-density training system (4 × 2 m) from May to July, which were significantly higher compared with the training systems of 4 × 1.5, 5 × 2.5 and 6 × 3 m (Figure 2A). In August, the SHD incidence values were similar in the training systems of 4 × 2, 5 × 2.5 and 6 × 3 m, which were significantly different from the values of the training system of 4 × 1.5 m. The SHD incidence values were the highest in the high-density training system (4 × 1.5 m) from September to November but these values were not significantly different from the other three training systems.

In 2018, the SHD incidence values were the highest in the high-density training system (4 × 1.5 m) in all assessed months with the exception of June when the highest values were reached in the training system of 5 × 2 m (Figure 2B). The SHD incidence values in the high-density training system (4 × 1.5 m) were significantly higher than the values in the training system of 6 × 3 m in May and September, and in the training systems of 4 × 2, 5 × 2.5 and 6 × 3 m in July and August. (Figure 2B). In October and November 2018, the SHD incidence values were not significantly different among the four training systems.

In 2019, the SHD incidence values were the highest in the high-density training system (4 × 1.5 m) in all assessed months (Figure 2C).The SHD incidence values in the high-density training system (4 × 1.5 m) were significantly higher than the values in the training system of 6 × 3 m in May, in the training systems of 5 × 2.5 and 6 × 3 m in June and August, and in the training systems of 4 × 2, 5 × 2.5 and 6 × 3 m in July (Figure 2C). In September, October and November 2019, the SHD incidence values were not significantly different among the four training systems.

#### 3.2.3. Cultivar ‘Stanley’

In the case of plum cultivar ‘Stanley’, SHD incidences were the highest in 2019 in the 4 × 1.5 m training system (ranging between 0 and 29.1%) and the lowest in 2017 in the 5 × 2.5 m training system (ranging between 0 and 4.3%, Figure 3).

In 2017, SHD progress began in mid-July in the training system of 4 × 1.5 m and in mid-September in the other three training systems (Figure 3A). The SHD progress of the four training systems started int mid-August in 2018, and in mid-June in 2019 (Figure 3). Following this, the disease increased with various progress speeds until the last assessment date (in November) in all years and in all training systems (Figure 3).

In 2017, the SHD incidence values were the highest in the mid training system (4 × 2 m) from September to November, which were significantly higher compared with the training system of 5 × 2.5 and 6 × 3 m in September and October, and in the training systems of 6 × 3 m in November (Figure 3A).

In 2018, the SHD incidence values were the highest in the high-density training system (4 × 1.5 m) in September, which were significantly different from the values in the training systems of 5 × 2.5 and 6 × 3 m (Figure 3B). The SHD incidence values were the highest in the mid-density training system (4 × 2 m) in October and November, which were significantly different from the values in the training systems of 5 × 2.5, and 6 × 3 m in September and October and in the training system of 6 × 3 m in November (Figure 3B).

In 2019, the SHD incidence values were the highest in the mid-density training system (4 × 2 m) in August, which were significantly different from all the other three training systems (Figure 3C). The SHD incidence values were the highest in the high-density training system (4 × 1.5 m) from September to November, which were significantly different from the values in the training systems of 5 × 2.5 and 6 × 3 m in September and in all the other three training systems (4 × 2, 5 × 2.5 and 6 × 3 m) in October and November (Figure 3C). In July 2019, the SHD incidence values were not significantly different among the four training systems.

#### 3.2.4. Cultivar ‘President’

In the case of plum cultivar ‘President’, SHD incidences were the highest in 2018 in the 4 × 1.5 m training system (ranging between 11.8 and 91.2%) and the lowest in 2017 in the 6 × 3 m training system (ranging between 0 and 65.3%, Figure 4).

SHD progresses in the four training systems started before the first assessment date (first decade of May) with the exceptions of the training systems of 5 × 2.5 and 6 × 3 m in 2017. Following this, the disease progressed continuously until the last assessment date (in November) in all years and in all training systems (Figure 4).

In 2017, the SHD incidence values were the highest in the high-density training system (4 × 1.5 m) in May and from August to November, which were significantly higher compared with the training systems of 4 × 2, 5 × 2.5, and 6 × 3 m in May, August and October, and the training systems of 5 × 2.5, and 6 × 3 m in September and November (Figure 4A). The SHD incidence values were the highest in the mid-density training system (4 × 2 m) in June, which were significantly different from the values of all the other three training systems (Figure 4A). The SHD incidence values were the highest in the low to mid density training system (5 × 2.5 m) in July, which were significantly different from the values of all the other three training systems (Figure 4A).

In 2018, the SHD incidence values were the highest in the high-density training system (4 × 1.5 m) from May to July and from October to November, which were significantly different from the training system of 5 × 2.5 in May, and from the training systems of 5 × 2.5 and 6 × 3 m in June, July, October and November (Figure 4B). The SHD incidence values were the highest in the mid-density training system (4 × 2 m) in August and September, which were significantly different from the values of the three training systems of 5 × 2.5 and 6 × 3 m (Figure 4B).

In 2019, the SHD incidence values were the highest in the high-density training system (4 × 1.5 m) from June to July and from October to November, which were significantly different from the training systems of 5 × 2.5 in June, July, October and November (Figure 4C). The SHD incidence values were no different from each other in the four training systems in May, August and September (Figure 4C).

### 3.3. Final Disease Incidence

Analyses of variance for the final disease incidences of SHD indicated significant (*p* < 0.05) differences amongst years, training systems and cultivars (Table 4). There were no significant interactions among the treatment factors.

According to the results of the ANOVA, the final disease incidences of SHD were shown separately for years, training systems and cultivars (Table 5). The values of the final disease incidence were 2 to 20 times lower on cv. ‘Stanley’ compared to the other three cultivars in all years, which was significantly different (*p* < 0.05). In general, the values of the final disease incidence increased in the order of high, mid, mid-low and low training systems.

The lowest final disease incidence value was 4.8% in the 5 × 2.5 m training system for cv. ‘Stanley’ in 2017, while the highest one was 100% for cv. ‘Bluefre’ in the training systems of 4 × 1.5, 4 × 2, and 5 × 2 m for cv. ‘Bluefre’ in 2017, and for all training systems in 2019 (Table 5). The overall years for the final disease incidences were significantly different only for cvs. ‘Stanley’ and ‘President’ when all cultivars were combined. Analyses of the overall training systems showed that the values of the final disease incidence in the training system of 4 × 1.5 m were significantly different from the training systems of 5 × 2.5 and 6 × 3 m when all years and all cultivars were combined (Table 5).

Analyses of each cultivar showed that the final disease incidence varied among training systems and years (Table 5). In case of cv. ‘Bluefre’, the values of the final disease incidence were not significantly affected by years and training systems. The final disease incidence values of the training system of 4 × 1.5 m were significantly different from the training systems of 4 × 2, 5 × 2.5 and 6 × 3 m, for cv. ‘Cacanska lepotica’ in 2018, and for cv. ‘Stanley’ in 2017 and 2019. The final disease incidence values of the training system of 4 × 1.5 m were significantly different from the training systems of 5 × 2.5 and 6 × 3 m, for cv. ‘Cacanska lepotica’ in 2017 and 2019, and for cv. ‘President’ in 2017 and 2018. The final disease incidence values of the training system of 4 × 1.5 m were significantly different from the training systems of 6 × 3 m, for cv. ‘Stanley’ in 2018, and for cv. ‘President’ in 2019.

### 3.4. AUDPC

Analyses of variance for the AUDPC values of SHD indicated significant (*p* < 0.001) differences amongst years, training systems and cultivars (Table 4). There were no significant interactions among the treatment factors.

According to the ANOVA, the AUDPC values of SHD were shown separately for years, training systems and cultivars (Table 6). The AUDPC values were the lowest for cv. ‘Stanley’ compared to the other three cultivars.

The lowest AUDPC value was 94 days^−1^ in the 5 × 2.5 m training system for cv. ‘Stanley’ in 2017, while the highest one was 15,001 days^−1^ for cv. ‘Bluefre’ in the training systems of 4 × 1.5 m in 2019 (Table 6). The AUDPC values for the overall years were significantly different for cvs. ‘Bluefre’, ‘Stanley’ and ‘President’ when all cultivars were combined. Analyses of the overall training systems showed that the AUDPC values of the training system of 4 × 1.5 m were significantly different from the training system of 6 × 3 m, when all years and all cultivars were combined (Table 6).

The AUDPC values varied among the training systems and years (Table 6) of each cultivar. The AUDPC values were not significantly affected by the training systems for cv. ‘Bluefre’ in 2017 and 2019 and for cv. ‘President’ in 2019. The AUDPC values of the training system of 4 × 1.5 m were significantly different from the training systems of 4 × 2, 5 × 2.5 and 6 × 3 m, for cv. ‘Cacanska lepotica’ in 2018, and for cv. ‘Stanley’ in 2017 and 2019. The AUDPC values of the training system of 4 × 1.5 m were significantly different from the training systems of 5 × 2.5 and 6 × 3 m, for cv. ‘Cacanska lepotica’ in 2019, and for cv. ‘President’ in 2018. The AUDPC values of the training system of 4 × 1.5 m were significantly different from the training systems of 5 × 2.5 m, for cv. ‘Cacanska lepotica’ in 2017. The AUDPC values of the training system of 4 × 1.5 m were significantly different from the training systems of 6 × 3 m, for cv. ‘Bluefre’ in 2018, for cv. ‘Stanley’ in 2018, and for cv. ‘President’ in 2017.

### 3.5. Time Periods When High vs. Low Density Training Systems and Cultivar Combination Can Reduce the Disease Development

**Figure 5:**
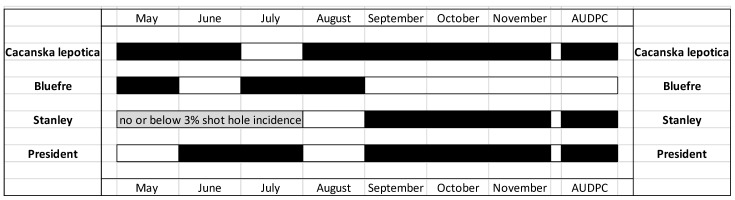
Time periods when high- vs. low-density training systems and cultivar combinations can reduce the disease development of shot hole disease incidence and area under the disease progress curve (AUDPC) in an integrated plum orchard at Debrecen-Pallag, Eastern Hungary. Low- and high-density training systems are 6 × 3 m and 4 × 1.5 m, respectively. Years were combined in the data analyses. Different color boxes represent significant differences at *p* = 0.05 level between low- and high-density training systems at a given month for the four plum cultivars. White color represents ‘no significant differences’ and black color represents ‘significant differences at *p* = 0.05 level. Grey box represents no or below 3% shot hole incidence for cv. ‘Stanley’.

High (4 × 1.5 m) vs. low (6 × 3 m) density training systems reduced the shot hole incidence and AUDPC in each of the assessed months depending on cultivar susceptibility to shot hole (Figure 5). The disease reduction effect of low vs. high training systems were various from May to August among the cultivars, and cv ‘Stanley’ showed no effect due to a low disease incidence (Figure 3). The low-density training system reduced AUDPC and SHD incidence consistently for three cultivars (‘Čačanska lepotica’, ‘Stanley’ and ‘President’) in September, October and November, compared to the high-density training system (Figure 5). Only cv. ‘Bluefre’ showed no effect either on disease incidence or AUDPC, due to a very high disease incidence in all training systems from September to November (Figure 2).

## 4. Discussion

In this study, we evaluated the effect of four training systems and four cultivars with various SHD susceptibility on shot hole temporal epidemics in an integrated plum orchard. In general, the SHD incidences and AUDPC of individual cultivars were lower on trees under the low (6 × 3 m) density training systems, compared to trees under the high (4 × 1.5 m) density training system, depending on the susceptibility of the cultivars and the annual weather conditions.

The results of this study showed a great annual variation in the SHD incidences of the evaluated four plum cultivars, which are in agreement with previous studies on various fruit species, e.g., [4,6,7,9,15,21,25,27,28,29,30,31]. Cultivars ‘Čačanska lepotica’ and ‘Bluefre’ showed high, SHD incidences, cv. ‘Bluefre‘ and ‘President’ showed mid-high SHD incidences, and ‘Stanley’ showed low SHD incidences (91–100%, 62–91%, and 8.4–30%, respectively) at harvest in all years, independently of a training system (Table 5). In agreement with the previous research of Benedek et al. [30], Romanazzi et al. [33], Bubici et al. [7] and Khromykh et al. [34], our results indicate that SHD resistance has a great influence on the disease’s development; therefore, the successful incorporation of the promising SHD-resistant plum cultivars into the growing practice is essential in those plum-growing areas where SHD is endemic. In addition to the SHD susceptibility of a plum’s genotype, the nitrogen and potassium content of the leaves [35] and the growth habits of the trees (e.g., a dense type of canopy or an open type of canopy) can also be factors that cause differences in the observed final SHD incidences [31]. Tutida et al. [35] showed that plum cultivars with a higher leaf content of nitrogen and potassium reduced SHD infections. In addition, plum trees with open canopies allowed better sunlight penetration and thus better photosynthetic activities in the canopy, compared to cultivars with dense canopies [31]. Moreover, variations in growth habit can also affect the spray depositions in the canopy which can influence the temporal dynamics of *S. carpophila* infection during the season.

The SHD incidences and/or AUDPC of the investigated cultivars were lower under the low (6 × 3 m) density training systems, compared to the high (4 × 1.5 m) density training system, except for AUDPC on cv. ‘Bluefre’ (Table 5 and Table 6; Figure 5). The effect of training systems on annual SHD progress was not previously evaluated in stone fruit orchards, but on similar diseases (such as leaf spot, which causes early leaf defoliation) that were studied in sweet cherry orchards [24]. Our results were in contrast with the study of Vámos and Holb [24] on sweet cherry vs. leaf spot pathosystems, as leaf spot incidences were lower in the higher density (4 × 1 m) orchard, compared to the lower density (5 × 2 m) one. Vámos and Holb [24] concluded that the depositions of spray droplets have better distribution within the tree canopy of the higher density orchard compared to the lower density one, which resulted in lower numbers of leaf spot infections during the season In this plum study, despite the larger trees being in the low (6 × 3 m) density training systems, they had a more open tree canopy (due to different pruning actions) compared to the high (4 × 1.5 m) density training system. This resulted in increasing sunlight penetration and air movement within the tree canopy of the low-density training system. Thus, not the tree volume but the airy component of the canopy may help to reduce SHD and enable better distributions of the spray droplets within the tree canopy.

Our study clearly demonstrated that the low-density training system reduced AUDPC and SHD incidence consistently for the cultivars with high or mid-high susceptibility to SHD, compared to the high-density training system. Low or no effects were seen on the low susceptibility cultivar (Figure 5). These results indicate that certain combinations of training systems and cultivars can significantly reduce the temporal development of SHD during the season and the accumulation of inoculum sources by the end of the season. This information may be successfully used for the most suitable selection of training system vs. cultivar combinations in those regions where SHD can cause severe epidemics. However, it is important to note that our results on SHD may need to be adjusted in regions with more humid and/or colder climates than Central Europe.

## 5. Conclusions

Our study showed that both training system and cultivar susceptibility can significantly influence the temporal epidemics of SHD in an integrated plum orchard. More specifically:

(i) Plum cultivars with high or mid-high susceptibility to SHD showed continuous SHD development from May to November, while cultivars with low susceptibility to SHD showed no symptoms until mid-summer and then progressed slowly until November.
(ii) The annual disease incidences and AUDPC of SHD on plum cultivars with high or mid-high susceptibility to SHD showed more sensitivity to training systems, compared to cultivars with low susceptibility to SHD.

Certain combinations of training system and cultivar can significantly reduce the temporal development of SHD during the season and the accumulation of inoculum sources (AUDPC) by the end of the season. This may be successfully used as a part of the integrated pest management approach during establishing new plantations.

## Figures and Tables

**Table 1:** Plum cultivar characteristics used in this study including pedigree, origin, susceptibility to shot hole disease (SHD) and harvest time in an integrated plum orchard at Debrecen-Pallag, East Hungary (2017–2019).

Cultivar	Origin	Pedigree	SHD Susceptibility	Harvest Time	Reference
‘Čačanska lepotica’	Serbia	‘Wangenheims Frühzwetsche’ × ‘Besztercei’	mid-high	End July–early August	[30,31]
‘Bluefre’	USA	‘Stanley’ × ‘President’	mid-high	End August–early September	[31]
‘Stanley’	USA	‘Ageni’ × ‘Grand Duke’	low	End August–early September	[31]
‘President’	UK	Developed by English breeders	mid	mid-September	[31]

**Table 2:** Fungicide spray programmes against shot hole disease on four plum cultivars (‘Čačanska lepotica’, ‘Bluefre’, ‘Stanley’ and ‘President’) in four training systems (4 × 1.5, 4 × 2, 5 × 2.5 and 6 × 3 m) in an integrated plum orchard at Debrecen-Pallag, East Hungary (2017–2019).

Date	Phenological Stage	Active Ingredients	Trade Name	Dosage
2017				
13 April	White flower bud	tebuconazole	Folicur Solo 25WG	1 L ha^−1^
19 April	Full bloom	captan	Orthocid 80WDG	2 kg ha^−1^
		tebuconazole	Folicur Solo	1 L ha^−1^
3 May	Fruit set	mancozeb	Manzate	0.2 L ha^−1^
20 May	Fruit swelling I.	dithianon	Delan	0.14 kg ha^−1^
15 June	Fruit swelling II.	prochloraz	Mirage 45 EC	450 g L^−1^
20 July	Fruit swelling III.	penconazole	Topas 100 EC	0.5 L ha^−1^
25 September	After harvest	copper hydroxide	Funguran-OH 50WP	0.1%
2018				
15 April	White flower bud	tebuconazole	Folicur Solo 25WG	1 L ha^−1^
21 April	Full bloom	tebuconazole	Folicur Solo	1 L ha^−1^
5 May	Fruit set	captan	Orthocid 80WDG	2 kg ha^−1^
24 May	Fruit swelling I.	dithianon	Delan	0.14 kg ha^−1^
18 June	Fruit swelling II.	penconazole	Topas 100 EC	0.5 L ha^−1^
24 July	Fruit swelling III.	prochloraz	Mirage 45 EC	450 g L^−1^
23 September	After harvest	copper hydroxide	Funguran-OH 50WP	0.1%
2019				
16 April	White flower bud	tebuconazole	Folicur Solo 25WG	1 L ha^−1^
22 April	Full bloom	captan	Orthocid 80WDG	2 kg ha^−1^
2 May	Fruit set	dithianon	Delan 75 WP	0.14 kg ha
22 May	Fruit swelling I.	mancozeb	Manzate	0.2 L ha^−1^
19 June	Fruit swelling II.	prochloraz	Mirage 45 EC	450 g L^−1^
26 July	Fruit swelling III.	penconazole	Topas 100 EC	0.5 L ha^−1^
26 September	After harvest	copper hydroxide	Funguran-OH 50WP	0.1%

**Table 3:** Analyses of variance for the effects of years (2017, 2018 and 2019); training systems (4 × 1.5, 4 × 2, 5 × 2.5 and 6 × 3 m); cultivars (‘Čačanska lepotica’, ‘Bluefre’, ‘Stanley’ and ‘President’); and assessment dates (days 0, 30, 60, 90, 120, 150 and 180) on incidences of shot hole disease in an integrated plum orchard at Debrecen-Pallag, East Hungary. Bold figures indicate significant differences at *p* < 0.05.

Source of Variance	df ^1^	MS ^2^	F ^3^	*p* ^4^
Year (Y)	2	1097.42	30.36	**<0.001**
Training system (T)	3	1175.60	32.52	**<0.001**
Main plot error	6	52.97	-	-
Cultivar (C)	3	48,526.6	1342.3	**<0.001**
T × C	9	137.79	3.81	0.0516
Sub-plot error	18	79.96	-	-
Assessment date (A)	6	23,385.8	646.88	<0.001
T × A	18	31.64	0.88	0.6094
C × A	18	52.98	1.47	0.1908
T × C × A	48	24.13	0.67	0.9251
Sub-sub plot error	108	36.15	-	-

^1^ df = degree of freedom. ^2^ MS = Mean squares. ^3^ F = F-tests. ^4^ *p* = Probability value.

**Table 4:** Analyses of variance for the effects of years (2017, 2018 and 2019); training systems (4 × 1.5, 4 × 2, 5 × 2.5 and 6 × 3 m); and cultivars (‘Čačanska lepotica’, ‘Bluefre’, ‘Stanley’ and ‘President’) on final disease incidence (*Y_f_*) and Area Under the Disease Progress Curve (AUDPC) of shot hole disease in an integrated plum orchard at Debrecen-Pallag, East Hungary. Bold figures indicate significant differences at *p* < 0.05.

Source of Variance	df ^1^	MS ^2^	F ^3^	*p* ^4^
*Final disease incidence—Y_f_*				
Year (Y)	2	40.27	3.56	**0.0498**
Training system (T)	3	284.81	25.17	**<0.001**
Main plot error	6	7.24	-	-
Cultivar (C)	3	14,875.2	1314.4	**<0.001**
T × C	9	178.42	42.5	0.0511
Sub-plot error	18	11.32	-	-
*AUDPC*				
Year (Y)	2	6,575,770	14.48	**<0.001**
Training system (T)	3	6,111,406	13.46	**<0.001**
Main plot error	6	322,002	-	-
Cultivar (C)	3	24,582,714	541.38	**<0.001**
T × C	9	727,494	1.61	0.1885
Sub-plot error	18	454,052	-	-

^1^ df = degree of freedom. ^2^ MS = Mean squares. ^3^ F = F-tests. ^4^ *p* = Probability value.

**Table 5:** The effects of years (2017, 2018 and 2019); training systems (4 × 1.5, 4 × 2, 5 × 2.5 and 6 × 3 m); and cultivars (‘Čačanska lepotica’, ‘Bluefre’, ‘Stanley’ and ‘President’) on final disease incidence (*Y_f_*) of shot hole disease in an integrated plum orchard at Debrecen-Pallag, East Hungary. Values within the given cultivar coupled with different letters are significantly different among each training system at *p* = 0.05 according to LSD *t*-tests; ns: nonsignificant.

Year/					Cultivar					
Training System	‘Čačanska l.’		‘Bluefre’		‘Stanley’		‘President’		Overall Cult.	
***2017***										
4 × 1.5	89.1	c	100	ns	15.2	b	80.1	b	71.1	b
4 × 2	84.2	bc	100	ns	7.3	a	73.5	ab	66.3	ab
5 × 2.5 s	64.4	a	100	ns	4.8	a	67.2	a	59.1	a
6 × 3	74.7	ab	99.0	ns	6.1	a	65.1	a	61.2	a
LSD_0.05_	11.7		-		3.1		10.8		8.7	
***2018***										
4 × 1.5	88.7	b	94.1	ns	19.4	b	91.2	b	73.4	b
4 × 2	71.0	a	97.1	ns	21.3	b	82.0	ab	67.9	ab
5 × 2.5 s	73.6	a	94.8	ns	18.3	b	78.4	a	66.3	ab
6 × 3	70.2	a	91.5	ns	14.0	a	75.2	a	62.7	a
LSD_0.05_	10.4		-		3.6		11.5		8.8	
***2019***										
4 × 1.5	85.1	b	100	ns	29.6	c	78.4	b	73.3	b
4 × 2	74.0	ab	100	ns	24.6	b	73.1	ab	67.9	ab
5 × 2.5 s	64.2	a	100	ns	17.1	a	72.0	ab	63.3	a
6 × 3	62.9	a	100	ns	14.3	a	71.5	a	62.2	a
LSD_0.05_	12.1		-		4.1		6.8		7.5	
***Overall years***										
2017	78.1	ns	99.8	ns	8.4	a	71.5	a	64.4	ns
2018	75.9	ns	94.4	ns	18.3	b	81.7	b	67.6	ns
2019	71.6	ns	100	ns	21.4	b	73.8	ab	66.7	ns
LSD_0.05_	-		-		3.6		9.8		-	
***Overall training***										
4 × 1.5	87.6	b	98.0	ns	21.4	b	83.2	b	72.6	b
4 × 2	76.4	a	99.0	ns	17.7	b	76.2	ab	67.3	ab
5 × 2.5 s	67.4	a	98.3	ns	13.4	a	72.5	a	62.9	a
6 × 3	69.3	a	96.8	ns	11.5	a	70.6	a	62.0	a
LSD_0.05_	11.1		-		4.1		10.7		7.4	

**Table 6:** The effects of the effects of years (2017, 2018 and 2019); training systems (4 × 1.5, 4 × 2, 5 × 2.5 and 6 × 3 m); and cultivars (‘Čačanska lepotica’, ‘Bluefre’, ‘Stanley’ and ‘President’) on the Area Under the Disease Progress Curve (AUDPC) of shot hole disease in an integrated plum orchard at Debrecen-Pallag, East Hungary. Explanations for LSD-test are given in Table 5.

Year/					Cultivar					
Training System	‘Čačanska l.’		‘Bluefre’		‘Stanley’		‘President’		Overall Cult.	
***2017***										
4 × 1.5	8985	b	10,900	ns	795	c	7440	b	6855	ns
4 × 2	8460	ab	12,660	ns	225	b	6435	ab	6945	ns
5 × 2.5 s	6060	a	11,730	ns	94	a	5865	ab	5936	ns
6 × 3	7020	ab	10,845	ns	150	ab	5175	a	5798	ns
LSD_0.05_	1834		-		87		1752		-	
***2018***										
4 × 1.5	10,230	b	11,400	b	1005	bc	9855	b	8123	b
4 × 2	7275	a	10,785	ab	1035	c	9240	ab	7084	ab
5 × 2.5 s	7845	a	10,590	ab	810	ab	7410	a	6664	ab
6 × 3	7170	a	8985	a	630	a	7245	a	6008	a
LSD_0.05_	1967		2271		192		2005		2101	
***2019***										
4 × 1.5	9105	b	15,001	ns	2535	c	7950	ns	8648	a
4 × 2	7800	ab	14,160	ns	1980	b	7005	ns	7736	ab
5 × 2.5 s	6630	a	13,860	ns	1275	a	7650	ns	7354	ab
6 × 3	6390	a	13,020	ns	1080	a	7185	ns	6919	b
LSD_0.05_	1894		-		462		-		1718	
***Overall years***										
2017	7631	ns	11,359	a	315	a	6229	a	6383	ns
2018	8130	ns	10,440	a	870	b	8438	b	6969	ns
2019	7481	ns	14,010	b	1718	c	7448	ab	7664	ns
LSD_0.05_	-		2311		441		2001		-	
***Overall training***										
4 × 1.5	9440	b	12,200	ns	1445	b	8415	b	7875	b
4 × 2	7845	ab	12,535	ns	1080	ab	7560	ab	7255	ab
5 × 2.5 s	6845	a	12,060	ns	725	a	6975	ab	6651	ab
6 × 3	6860	a	10,950	ns	620	a	6535	a	6241	a
LSD_0.05_	1932		-		452		1863		1602	
